# Regulation of Fc Receptor Endocytic Trafficking by Ubiquitination

**DOI:** 10.3389/fimmu.2014.00449

**Published:** 2014-09-18

**Authors:** Rosa Molfetta, Linda Quatrini, Francesca Gasparrini, Beatrice Zitti, Angela Santoni, Rossella Paolini

**Affiliations:** ^1^Department of Molecular Medicine, “Sapienza” University of Rome, Rome, Italy; ^2^Lymphocyte Interaction Laboratory, London Research Institute, Cancer Research UK, London, UK; ^3^Institute Pasteur-Fondazione Cenci Bolognetti, “Sapienza” University of Rome, Rome, Italy

**Keywords:** Fc receptors, ubiquitination, endocytosis, endocytic adaptors, innate immune cells

## Abstract

Most immune cells, particularly phagocytes, express various receptors for the Fc portion of the different immunoglobulin isotypes (Fc receptors, FcRs). By binding to the antibody, they provide a link between the adaptive immune system and the powerful effector functions triggered by innate immune cells such as mast cells, neutrophils, macrophages, and NK cells. Upon ligation of the immune complexes, the downstream signaling pathways initiated by the different receptors are quite similar for different FcR classes leading to the secretion of preformed and *de novo* synthesized pro-inflammatory mediators. FcR engagement also promotes negative signals through the combined action of several molecules that limit the extent and duration of positive signaling. To this regard, ligand-induced ubiquitination of FcRs for IgE (FcεR) and IgG (FcγR) has become recognized as a key modification that generates signals for the internalization and/or delivery of engaged receptor complexes to lysosomes or cytoplasmic proteasomes for degradation, providing negative-feedback regulation of Fc receptor activity. In this review, we discuss recent advances in our understanding of the molecular mechanisms that ensure the clearance of engaged Fcε and Fcγ receptor complexes from the cell surface with an emphasis given to the cooperation between the ubiquitin pathway and endosomal adaptors including the endosomal sorting complex required for transport (ESCRT) in controlling receptor internalization and sorting along the endocytic compartments.

## Introduction

Upon antigen recognition, immunoglobulins can bind via their Fc tail to Fc receptors (FcRs), which are expressed by many different cell types and particularly phagocytes.

Most FcRs including receptors for IgA (FcαRI), IgE (FcεRI), and IgG (FcγRI, FcγRIIA, FcγRIIIA, and FcγRIV) are activating receptors (1, 2). They comprise high-affinity receptors (FcαRI, FcεRI, FcγRI), that bind monomeric immunoglobulins and low-affinity receptors (FcγRIII and FcγRIV in the mouse, and FcγRIIA and FcγRIIIA in humans) that only interact with antibodies in the form of immune complexes. This ligation can trigger numerous cellular effector functions including phagocytosis, antibody-dependent cellular cytotoxicity (ADCC), and secretion of cytokines or other inflammatory mediators (3).

With the exception of human FcγRIIA, the activating FcRs are multi-chain receptors composed by a ligand-binding α subunit and one or more transducing subunit(s) containing in the intracytoplasmatic domain(s), the immunoreceptor tyrosine-based activatory motif (ITAM).

Upon FcR crosslinking, the signaling pathways propagated by the activating receptors are quite similar for different FcR classes, and initiate with ITAM tyrosine phosphorylation by kinases of the Src family. Then, phosphorylated ITAMs lead to the recruitment of Syk-family kinases, followed by activation of various downstream targets, such as the linker for activation of T cells (LAT). Once phosphorylated, LAT recruits the phospholipase Cγ that hydrolyzes the membrane phosphatidyl inositol 4,5-bisphosphate [PtdIns(4,5)P_2_] to form the soluble inositol 1,4,5-trisphosphate (IP_3_) and the membrane bound diacylglycerol. These second messengers increase intracellular calcium level and trigger further downstream signaling.

Besides calcium-dependent pathways, the Ras- and Raf-MAPK (mitogen-activated protein kinase) cascades are also triggered following FcR crosslinking.

Several surface inhibitory receptors are able to counteract FcR-mediated responses. They share the presence in their cytoplasmic tail of an immunoreceptor tyrosine-based inhibitory motif (ITIM) that, once phosphorylated, recruits and activates lipid and protein phosphatases [i.e. SH2 domain-containing inositol-polyphosphate 5-phosphatase (SHIP) and SH2 domain-containing protein-tyrosine phosphatase (SHP)] (4). Among the FcR family, the only known ITIM-containing receptor is the low-affinity receptor for IgG, referred to as FcγRIIB, which can be expressed into two different isoforms on all cells of the immune system, with the exception of T and NK cells (2, 5).

To exert its inhibitory properties, FcγRIIB must be co-aggregated with activating receptors by the same immune complex at the cell surface (6).

Regulation of FcR-dependent cell activation also includes different inhibitory mechanisms originated by activating FcRs themselves (7–11).

Some ITAM-containing FcRs, such as FcαRI and FcγRIIIA that are associated with the common γ chain, can generate negative signals able to affect positive signals delivered by other activating FcRs co-expressed on the same cell (9, 10). The activating versus inhibitory ITAM configuration is mostly dependent on how the ITAM-containing receptor interacts with its ligand. In the case of FcαRI, multimeric interactions induce an activating signal, whereas interactions of the receptor with ligands binding at low valency, such as monomeric IgA, generate an inhibitory response (9). This latter response involves the action of the phosphatase SHP-1 and requires the formation of intracellular inhibisome clusters containing the targeted activating receptor.

Other ITAM-containing FcRs, such as FcεRI, can trigger both activating and inhibitory signals, thus contributing to their own, autonomous control (7, 11). In this case, the negative signal involves the coordinated action of adaptors (12–14), phosphatases (15, 16), and ubiquitin ligases (17–19) that limit the intensity and duration of positive signals, thus modulating cellular functions.

A negative-feedback regulation essential for the precise control of cellular functions is also provided by the internalization and degradation of activated FcR complexes (20–22).

Multiple endocytic pathways can potentially mediate the ligand-induced Fc receptor internalization through the action of adaptor proteins that recognize specific signals present in the cytoplasmic tails of the plasma membrane proteins (23, 24).

Several recent evidences pointed to FcR ubiquitination as an important additional signal for membrane protein endocytosis (22).

This review overviews the role of the ubiquitin pathway and the endosomal sorting complex required for transport (ESCRT) machinery in the regulation of Fcε and Fcγ receptor internalization and sorting along the endocytic compartments.

How this pathway regulates Fc receptor-mediated neutralization of intracellular immune complexes will be also briefly discussed.

## The Ubiquitin Pathway

Ubiquitination is a post-translational modification in which the small conserved peptide ubiquitin (Ub) is covalently attached to the ε-amino group of lysine (K) residues of target proteins (25, 26).

The Ub-conjugating reaction, termed ubiquitination, is catalyzed by the successive action of three classes of enzymes. The Ub-activating enzyme (E1) forms a thiol-ester bond with the carboxy-terminal glycine of Ub in an ATP-dependent reaction. Activated Ub is, then, transferred to the Ub-conjugating enzyme (E2) by transthiolation, and finally conjugated to the substrate through the action of the Ub protein ligase (E3). This latter class of enzymes is responsible for substrate recognition and Ub ligation to the target protein, thus providing specificity to the Ub system. Like protein phosphorylation, ubiquitination is reversible. Indeed, several Ub-specific proteases, referred to deubiquitinating enzymes (DUBs), can cleave Ub from its target.

Proteins can be modified by the addition of a single molecule of Ub to a single K residue (monoubiquitination) or to different residues (multiubiquitination). These modifications regulate several cellular functions including virus budding, nuclear shuttling, transcription, and endocytosis (27, 28). Moreover, Ub moieties are often added to the target protein in the form of polyubiquitin chain (29). Indeed, Ub contains seven amino groups that can be used for chain formation *in vivo* (30), and different topologies of polyUb chains are associated with diverse biological functions (31). For instance, polyUb chains of at least four Ub molecules linked via K48, direct degradation of the target protein by the 26S proteasome (32), whereas K63-linked chains participate in several other cellular processes ranging from DNA damage repair to endocytosis (29, 33).

In regard to ubiquitination as a modification that generates a signal for endocytosis, several observations suggest that, although monoubiquitination is sufficient for the internalization and endosome-to-lysosome trafficking of plasma membrane proteins in both yeast and mammalian cells (34, 35), multiubiquitination and K63-linked polyubiquitination lead to a higher rate of endocytosis/lysosomal transport than monoubiquitination (36–38).

## Ubiquitin as an Endocytosis Signal of Membrane Receptors

The early endosomes, also known as “sorting tubular endosomes,” represent the first compartment deputed to receive incoming vesicles from plasma membrane. In fact, they accept cargoes destined to alternative fates, either recycling to the plasma membrane or endocytic sorting into the intraluminal vesicles (ILVs) of the multivesicular body (MVB) and the lysosomes responsible for cargo degradation. Those two alternative fates may depend on which route the cargo utilizes to enter into the cell and/or which signal the cargo presents.

Regarding the action of Ub as sorting signal, the first compelling evidence came from studies on yeast showing that ubiquitination of cell-surface proteins, such as G-protein-coupled receptors and transporters, is required for their vacuolar/lysosomal degradation (34, 39).

Studies in mammalian cells helped to support a general model in which Ub acts as a sorting signal (40). In such a model, ubiquitinated membrane proteins must be recognized by different endosomal molecular adaptors to be properly delivered to lysosomes for degradation.

The best-studied Ub-dependent routes are the clathrin-dependent endocytosis and the ESCRT-dependent sorting into MVBs.

Ubiquitinated proteins undergo Ub-dependent internalization mainly through clathrin-coated pits (36, 41–43).

The internalization process involves the action of several clathrin-binding adaptors that contain Ub-interacting motif (UIM) used to specifically recognize the ubiquitinated receptor (44). Among UIM-containing adaptors involved in endocytosis, the best characterized are Eps15 and Epsin (45–48). Knockdown of either Eps15 or Epsin as well as overexpression of mutant Eps15 or Epsin lacking UIMs inhibits Ub-dependent cargo internalization (43, 46, 48, 49).

Notably, even cargoes that do not require the Ub pathway for delivery to early endosomes need Ub as a signal for incorporation into the MVBs through the action of the ESCRT machinery (50–52).

The ESCRT machinery comprises four main distinct complexes (ESCRT-0, -I, -II, and -III) and several accessory components that recognize and deliver ubiquitinated membrane proteins into ILVs within MVB, which ultimately fuse with lysosomes.

The upstream complexes ESCRT-0, -I, and -II contain Ub-binding domains that are responsible for interactions with ubiquitinated cargoes. ESCRT-0 consists of only two subunits, the hepatocyte growth factor-regulated tyrosine kinase substrate (Hrs) and the signal transducing adaptor molecule (STAM), and forms large domains of clustered ubiquitinated cargoes, thanks to its polyvalent Ub-binding ability and its known participation in flat clathrin coats in early endosomes (53). Moreover, Hrs is also able to bind to the endosomally enriched lipid phosphatidylinositol 3-phosphate (PI3P), allowing the recruitment of the entire ESCRT-0 complex to early endosomes (54, 55).

Endosomal sorting complex required for transport-I and -II are heteromeric complexes not stably associated with endosomes, but able to interact with each other and with ESCRT-0 (56). They are mainly responsible for membrane budding into the lumen of the MVB (57, 58).

Endosomal sorting complex required for transport-III consists of four core and several accessory subunits that once correctly assembled are responsible for the detachment of membrane buds into the lumen of the MVB (59, 60). The complex has no Ub-binding domains, but instead actively recruits DUBs to remove Ub from the cargo before incorporation into the lumen of MVB.

Thus, the ESCRT machinery performs three different but related functions: recognition of ubiquitinated cargoes; endosomal membrane deformation allowing cargo to be sorted into endosomal invaginations; membrane abscission allowing the release of ILVs that contain the sorted cargoes.

Notably, several subunits of the ESCRT machinery itself become ubiquitinated/deubiquitinated. However, it is still unclear whether these modifications could provide a switch between active and inactive forms.

## The Ubiquitin Pathway as Endocytic Route of Fc Receptors

Among the different classes of FcRs, a role for Ub as a sorting signal has been formally demonstrated only for the high-affinity IgE receptor, FcεRI, and for the low-affinity IgG receptors, FcγRIIA and FcγRIIIA.

### FcεRI

FcεRI is constitutively expressed on the surface of mast cells and basophils as a heterotetramer composed by an IgE-binding α subunit, a four transmembrane-spanning ITAM-containing β subunit, and two identical disulfide-linked ITAM-containing γ subunits (61). Upon the ligation of multivalent antigen to FcεRI-bound IgE molecules, the receptor complex transduces intracellular signals leading to the release of preformed and *de novo* synthesized pro-inflammatory mediators that cause immediate anaphylactic reactions or prolonged allergic inflammation (62–64).

A negative-feedback regulation of FcεRI activity is provided by receptor ubiquitination that represents an important signal for the internalization and delivery of engaged receptor complexes to lysosomes for degradation.

The rat basophilic leukemia cell line namely RBL-2H3 has been widely used as an *in vitro* model since it retains many characteristics of mucosal mast cells, including the surface expression of ~300,000 FcεRI molecules (65). Early studies performed on RBL-2H3 cells (66, 67) have shown that FcεRI β and γ subunits are subjected to ubiquitination upon stimulation with IgE and multivalent antigen. Upon receptor engagement, both FcεRI subunits co-localize with the E3 Ub-ligase c-Cbl into lipid rafts (68) suggesting the involvement of c-Cbl in receptor ubiquitination.

Our group has, indeed, identified c-Cbl as the main E3 ligase responsible for antigen-induced receptor ubiquitination in RBL-2H3 cells (69).

We have more recently demonstrated that FcεRI β and γ subunits are monoubiquitinated by c-Cbl at multiple sites upon antigen stimulation, and provided evidence that this modification controls receptor internalization (70).

Regarding the route(s) involved in FcεRI internalization upon antigen stimulation, several studies suggest that engaged receptors are internalized via clathrin-dependent endocytosis. Although FcεRI internalization does not require *de novo* formation of clathrin-coated pits (71), early morphological studies demonstrated that cross-linked FcεRI co-localized with clathrin-coated pits (72, 73), supporting the conclusion that receptor endocytosis is mainly clathrin-mediated.

Accordingly, we have reported that a rapid and efficient endocytosis of cross-linked FcεRI complexes required the presence of the adaptor CIN85 (Cbl-interacting protein of 85 kDa) (74), which is constitutively bound to endophilin, a regulatory component of clathrin-coated pit. However, two independent groups have more recently reported that siRNA-mediated clathrin depletion does not affect antigen-dependent FcεRI internalization (65, 75), demonstrating that engaged FcεRI complexes can efficiently take an alternative route for endocytosis if the clathrin pathway is inhibited.

To this regard, the implication of a lipid raft environment in regulating antigen-induced FcεRI endocytosis has been also envisaged (70, 75, 76). Fattakhova and co-authors have firstly reported that internalized FcεRI complexes remain associated with detergent insoluble structures (75). Moreover, it was subsequently showed that engaged FcεRI complexes internalized together with the raft-associated ganglioside GM1 (76), and that cholesterol depletion decreased antigen-induced FcεRI internalization (70).

We further evaluated the relationship between lipid rafts and FcεRI ubiquitination. Our results demonstrated that the recruitment of FcεRI subunits into lipid rafts precedes their ubiquitination, and the integrity of these microdomains is required for receptor ubiquitination (70). Most importantly, we have also shown a strong interdependence between lipid rafts and FcεRI endocytosis.

Thus, a cohesive model is one in which lipid rafts may drive alternative routes of receptor internalization in the absence of clathrin. Moreover, a cross-talk between clathrin-dependent and lipid raft-dependent endocytic routes may also be envisaged in mast cells. Indeed, proximity between clathrin and lipid rafts has been described in a morphological study (72), and the presence of clathrin in lipid raft enriched fractions was subsequently reported (75).

Regardless of the main endocytic route (clathrin-dependent versus lipid raft-dependent) involved, the UIM-containing adaptor proteins Eps15, Eps15R, and epsin, coupling ubiquitinated cargoes with components of the budding vesicles (45, 46), can regulate the endocytic trafficking of ubiquitinated FcεRI complexes. Although individual depletion of Eps15, Eps15R, and epsin failed to affect FcεRI entry in early endosomes, their simultaneous depletion impaired ligand-induced receptor endocytosis in RBL-2H3 cells (70), suggesting overlapping functions of these adaptors in regulating the uptake of ubiquitinated FcεRI complexes. This finding is consistent with a recent published result showing that overexpression of a dominant negative Eps15–DIII–GFP fusion protein only partially blocks antigen-dependent FcεRI internalization (65).

Among the different ESCRT complexes, ESCRT-0, and in particular Hrs, is considered as the best candidate for endosomal Ub-sorting receptor. Indeed, studies in mammalian cells demonstrate that loss of the Ub-binding domain of Hrs (a UIM variant that has two Ub-binding surfaces) disrupts Hrs ability to bind to ubiquitinated proteins and to retain Ub receptors on endosomes (55, 77, 78). Notably, we have observed that Hrs depletion sequesters ubiquitinated receptors into early endosomes and partially prevents their sorting into lysosomes for degradation (70), demonstrating a key role for Hrs in regulating the fate of internalized FcεRI complexes.

Moreover, we found that Hrs itself undergoes covalent modifications affecting its function as endocytic adaptor. Indeed, we demonstrated that Hrs is subjected to antigen-dependent tyrosine phosphorylation and monoubiquitination upon FcεRI engagement, and we identified Syk as the main kinase regulating both inducible Hrs post-translational modifications in RBL-2H3 cells (79). Moreover, by siRNA knock down of c-Cbl and complementary overexpression studies, we demonstrated that Hrs monoubiquitination is under the control of c-Cbl ligase activity, and that monoubiquitinated forms of Hrs, known to prevent Hrs ability to bind to ubiquitinated cargo (80), are preferentially confined on cytosolic fractions. On the contrary, an increase of Hrs phosphorylation was reproducibly observed only in membranes.

Our finding suggests that Hrs may need to be tyrosine phosphorylated to interact with other ESCRT components in order to ensure an efficient transport of ubiquitinated cargos to MVBs. The removal of monoubiquitinated Hrs from endosomal membrane could then facilitate the clearance of the non-functional adaptor and its replacement with non-ubiquitinated and sorting-competent Hrs.

**Figure 1:**
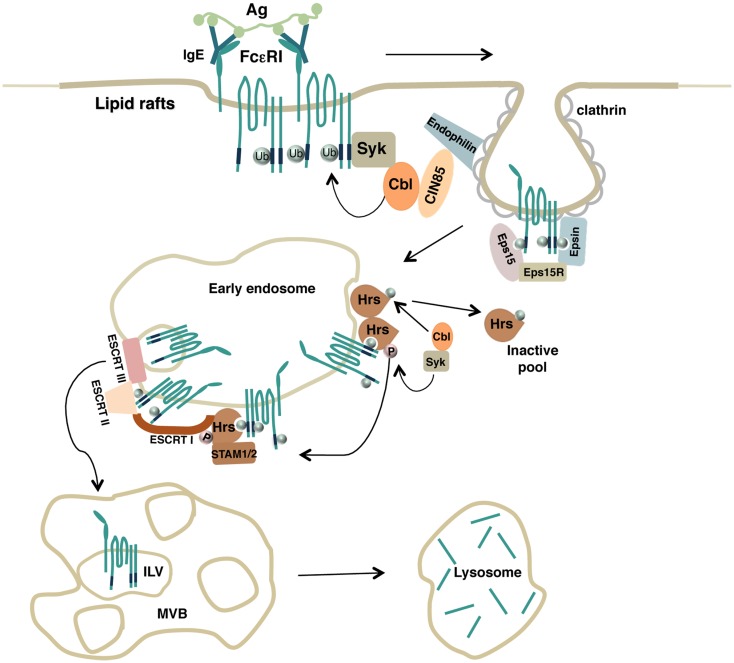
**Model depicting how FcεRI ubiquitination drives endocytosis of engaged receptor complexes**. Upon antigen stimulation, lipid rafts serve as a platform to recruit engaged FcεRI complexes and the Ub-ligase Cbl that promotes receptor multiubiquitination. Ubiquitinated receptors are then recognized by endocytic adaptors containing Ub-interacting motifs that drive both FcεRI clearance from the plasma membrane (Eps15, Eps15R, Epsin) and receptor sorting along the endosomal compartments (Hrs). Hrs itself becomes a substrate for Syk and Cbl enzymatic activities. Monoubiquitinated Hrs is removed from endosomal sorting sites whereas phosphorylated Hrs interacts with other endocytic adaptors of the ESCRT complexes to ensure the transport of ubiquitinated FcεRI complexes into the intraluminal vesicles (ILVs) of the multivesicular body (MVB) and to the lysosomes for degradation.

Overall, these results firstly support a key role for the Ub pathway and the ESCRT machinery to ensure endocytic trafficking of an Fc receptor to the lysosomes where degradation of the complexes can take place (Figure 1).

A role for Ub as a sorting signal in human mast cells and basophils has not been investigated yet.

### FcγRIIA

FcγRIIA is a single chain transmembrane receptor containing both a ligand-binding extracellular domain and an ITAM-like motif in its cytoplasmic tail responsible for signal transduction. This receptor is widely expressed on hematopoietic cells, and its engagement triggers many biological functions including degranulation and synthesis of cytokines (2). Moreover, FcγR crosslinking on phagocytes promotes endocytosis of small immune complexes and phagocytosis of large IgG-opsonized particles. These two internalization processes differ markedly in terms of molecular mechanisms involved: endocytosis is clathrin- and dynamin-dependent and does not require the integrity of cytoskeleton; phagocytosis involves assembly of F-actin and phosphatidylinositol 3-kinase activity (81). By following the internalization of FcγRIIA expressed in a CHO-derived cell line bearing a temperature-sensitive mutation in the E1 enzyme (CHO-ts20), Booth and coworkers provided the first evidence that the Ub machinery is required for endocytosis of soluble immune complexes by FcγRIIA, but it is dispensable for actin-driven phagocytosis of large antibody-coated particles (82). Elimination of the K residues present in the tail of FcγRIIA impaired endocytosis but did not affect either phagocytosis or phagosome maturation. Moreover, it was found that FcγRIIA is mainly modified by the addition of polyUb chains (83), although the specific K residue(s) involved in polyUb chain formation have not been identified yet.

The same group subsequently showed that Src family kinase-mediated tyrosine phosphorylation of FcγRIIA is not required for receptor endocytosis and ubiquitination of soluble immune complexes on COS-1 stable FcγR transfectants, but it is necessary for phagocytosis (83, 84). This finding was confirmed in freshly isolated human monocytes: inhibition of Src, Syk, and PI3K kinase activities affects FcγR phagocytosis of large opsonized particles without altering endocytosis of small immune complexes (84). Conversely, in human neutrophils, FcγRIIA ubiquitination, achieved by antibody-mediated receptor crosslinking, was found to be Src kinase-dependent (85).

**Figure 2:**
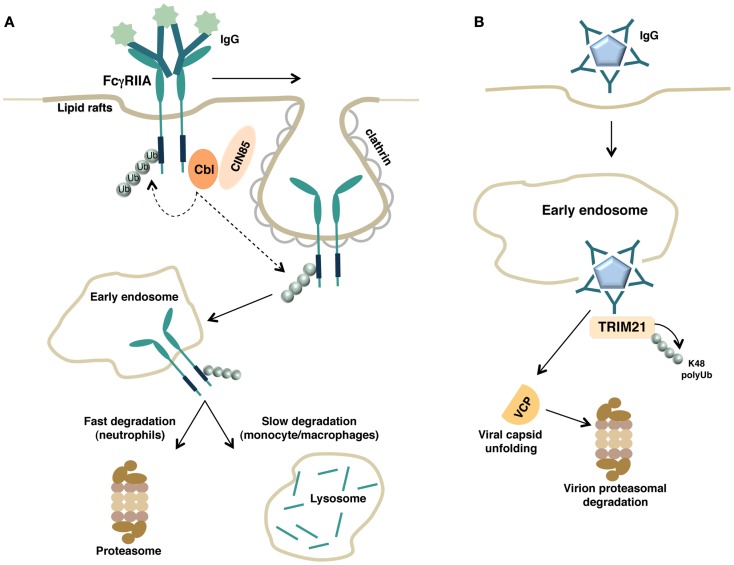
**(A)** Schematic representation of FcγRIIA down-regulation by the ubiquitin pathway is shown. Upon binding with soluble immune complexes, FcγRIIA undergoes clathrin-dependent internalization, Cbl-mediated polyubiquitination, and proteasomal or lysosomal degradation depending on the cellular context. **(B)** Intracellular neutralization of antibody-coated virions by TRIM21. Antibody-coated virions are internalized, released into the cytosol after escaping from the endosome, and detected by the cytosolic intracellular Fc receptor TRIM21. TRIM, acting as E3 ligase, catalyzes its own polyubiquitination allowing the recruitment of the ATPase p97/valosin-containing protein (VCP). VCP promotes the initial viral capsid unfolding, thus enabling the subsequent proteasomal degradation of the virion.

Thus, the contribution of phosphorylation events in regulating FcγRIIA ubiquitination appears to be different depending on the cellular context and experimental conditions. However, a common molecular mechanism regulating FcγRIIA down-modulation relies on the requirement of receptor ubiquitination and clathrin (Figure 2A). Accordingly, the down-regulation of FcγRIIA expression in human neutrophils is positively regulated by the clathrin adaptor protein CIN85 (86), as previously reported for FcεRI (74).

Regarding the role of lipid rafts, Barabè and colleagues demonstrated that the integrity of lipid rafts is required for a proper degradation of cross-linked FcγRIIA (87). Recruitment of engaged FcγRIIA and c-Cbl into lipid rafts is followed by Cbl-dependent receptor ubiquitination and degradation (85). Whether receptor ubiquitination occurs at the plasma membrane or later on during the transport into early endosomes has not been clarified yet (Figure 2A).

Concerning the main cellular location (cytosolic proteasomes versus lysosomes) implicated in FcγRIIA degradation, different results have been reported depending on the cellular context.

Proteasome but not lysosomal inhibitors prevent the rapid (few minutes upon receptor ligation) receptor degradation observed in human neutrophils (85), suggesting that ubiquitinated FcγRIIA itself may become a target for proteasomal degradation. Conversely, in monocyte-derived human macrophages, antibody-cross-linked FcγRIIA complexes are sorted into a lysosomal compartment within 60 min of receptor engagement (88), indicating that lysosomes may also contribute in FcγRIIA degradation (Figure 2A).

However, a role for UIM-containing adaptors and ESCRT complexes in FcγRIIA endocytic trafficking to the lysosomal compartment has not been investigated yet.

### FcγRIIIA

FcγRIIIA (type III receptor for IgG; CD16) is a multimeric receptor composed of a ligand-binding α chain associated with ITAM-containing γ and/or ζ dimers, initially identified as components of the FcεRI and the TCR, respectively (1). It binds to the Fc portion of human IgG1 and IgG3, is highly expressed on the cytotoxic CD56^dim^CD16^+^ NK cell subset, and mediates ADCC and cytokine/chemokine release.

FcγRIIIA down-regulation on human NK cells is a consequence of both metalloprotease-induced shedding and internalization of cross-linked receptors, and requires an intact actin cytoskeleton (89–93) and a clathrin-dependent pathway (Molfetta, unpublished observations).

Notably, we have shown that the FcγRIIIA ζ subunit undergoes Ub modification upon receptor aggregation in a phosphorylation-dependent manner (94), and suggested a role for ubiquitination in driving ζ chain lysosomal degradation, as previously reported for the same subunit in the context of the TCR/CD3 complex (95). Moreover, proteasome inhibition also impairs FcγRIIIA ζ chain degradation (Molfetta et al., unpublished observations), suggesting a cooperation of proteasomal and lysosomal degradative pathways.

Furthermore, engaged receptor complexes accumulate into lipid rafts (92), thus the contribution of a lipid raft environment in regulating FcγRIIIA ubiquitination can be also envisaged, as formally demonstrated for the FcεRI in mast cells and for the FcγRIIA on human neutrophils.

## The Ub Pathway and the Fc Receptor TRIM21: A New Mechanism for Virus Neutralization

Mallery and colleagues have recently discovered that antibodies, in addition to their extracellular activities, provide protection also inside cells mediating an intracellular immune response termed antibody-dependent intracellular neutralization (96). Antibodies that bind to non-enveloped virus before infection remain attached to the viral particle and are carried into the cell. Antibody-coated virions are then released into the cytosol after escape from the endosome and detected by a cytosolic intracellular Fc receptor called tripartite motif-containing 21 (TRIM21), which binds to IgG with high affinity (97). TRIM21 also possesses a RING domain with E3 ubiquitin ligase activity, and through its enzymatic activity, it targets the virion for proteasomal degradation. Interestingly, by performing *in vitro* ubiquitination assay, the authors found that TRIM21 forms K48 Ub chain only on itself (96), suggesting that recruitment to the proteasome is not dependent on direct ubiquitination of either the antibody or the virus, but rather autoubiquitination of the Fc receptor. Although the precise molecular mechanism of TRIM-mediated viral degradation *in vivo* is currently unclear, the same group subsequently reported that the presence and activity of the ATPase p97/valosin-containing protein (VCP), an enzyme with Ub-selective segregase and unfoldase activity, is required for the disassembly and the partial unfolding of the virion allowing its subsequent proteasomal degradation (98).

A proposed model of TRIM-mediated viral neutralization and degradation is depicted in Figure 2B.

## Concluding Remarks

As discussed above, ligand-induced ubiquitination of FcRs has become recognized as a modification that ensures recognition, internalization, and/or delivery of engaged receptor complexes to cytosolic proteasomes or lysosomes for degradation, thus down-regulating Fc receptor-transduced signaling.

In particular, FcεRI ubiquitination provides a signal for both internalization and delivery of engaged receptor complexes to lysosomes for degradation. In the case of FcγRIIA, Ub-dependent receptor down-regulation appears to be a common feature upon binding with soluble immune complexes in both monocytes and neutrophils. However, the very rapid proteasomal-dependent FcγRIIA degradation observed in neutrophils may represent a unique feature of these cells. In this scenario, the use of the proteasomal pathway for FcγRIIA down-regulation could avoid an excessive activation of neutrophils ensuring a non-inflammatory clearance of immune complexes. Conversely, in monocytes, a slower kinetics of endocytosis can guarantee FcγRIIA sorting along the endocytic machinery and delivery to lysosomes. Future challenges in the field will be to understand the contribution of UIM-containing adaptors and the ESCRT machinery in driving FcγRIIA endocytic sorting, since information is presently available only for FcεRI.

In regard to FcγRIIIA, it will be important to understand whether the proteasome is directly responsible for degradation of ubiquitinated receptor itself and/or promotes degradation of other ubiquitinated substrates that control receptor endocytosis and intracellular sorting.

Another major question is whether ubiquitination could contribute to the down-regulation of other FcRs in particular, those able to trigger either activating or inhibitory signals, such as FcαRI.

As also briefly discussed above, ubiquitination of the cytosolic Fc receptor TRIM21 regulates a new intracellular process responsible for neutralization of antibody-bound pathogens.

Interestingly, the Ub-ligase activity of TRIM21, in addition to target viruses for destruction, also alerts the body to infection. Indeed, upon recognition of antibody-coated virions, TRIM21 catalyzes the formation of free K63-linked Ub chains, which activate several transcription factors inducing a potent antiviral state (99).

## Conflict of Interest Statement

The authors declare that the research was conducted in the absence of any commercial or financial relationships that could be construed as a potential conflict of interest.
